# Development of the National Strategy for Quality of Care and Patient Safety for Greece: co-creation process and lessons learnt

**DOI:** 10.1093/intqhc/mzaf135

**Published:** 2025-12-30

**Authors:** Válter R Fonseca, Damir Ivanković, Constantina Vasileiou, Marie Stridborg, Christos Triantafyllou, Thanos Myloneros, Vion Psiakis, Zoi Tsimtsiou, Vasiliki Kapaki, Daphne Kaitelidou, Lilian Vildiridi, João Breda

**Affiliations:** WHO Athens Quality of Care and Patient Safety Office, World Health Organization Regional Office for Europe, Athens, Greece; WHO Athens Quality of Care and Patient Safety Office, World Health Organization Regional Office for Europe, Athens, Greece; WHO Athens Quality of Care and Patient Safety Office, World Health Organization Regional Office for Europe, Athens, Greece; WHO Athens Quality of Care and Patient Safety Office, World Health Organization Regional Office for Europe, Athens, Greece; WHO Athens Quality of Care and Patient Safety Office, World Health Organization Regional Office for Europe, Athens, Greece; WHO Athens Quality of Care and Patient Safety Office, World Health Organization Regional Office for Europe, Athens, Greece; WHO Athens Quality of Care and Patient Safety Office, World Health Organization Regional Office for Europe, Athens, Greece; Department of Hygiene, Social-Preventive Medicine and Medical Statistics, School of Medicine, Aristotle University of Thessaloniki, Thessaloniki, Greece; Agency for Quality Assurance in Health S.A., Athens, Greece; Agency for Quality Assurance in Health S.A., Athens, Greece; Ministry of Health, Athens, Greece; WHO Athens Quality of Care and Patient Safety Office, World Health Organization Regional Office for Europe, Athens, Greece

**Keywords:** Greece, quality of care, National Strategy, healthcare reform, improvement science, leadership and management, person-centred care, patient safety, quality improvement, education and research

## Abstract

**Background:**

Improving the quality of care and patient safety is a global health priority. Before 2025, Greece lacked a comprehensive national strategy to address longstanding challenges such as fragmented service delivery, uneven care quality, and limited safety reporting. In 2023–2024, a multi-stakeholder initiative, led by the Ministry of Health, in collaboration with the WHO Regional Office for Europe, the Agency for Quality Assurance in Health, and the European Commission, supported the development of Greece’s first National Strategy for Quality of Care and Patient Safety (2025–2030).

**Methods:**

A multi-method, co-creation approach was adopted, combining a scoping review, a nationwide needs assessment (405 survey responses and 14 interviews), seven regional workshops (348 participants), and a validation process. Over 750 stakeholders, including policymakers, professionals, patients, and academics, were engaged in shaping the strategy.

**Results:**

Findings highlighted governance fragmentation, limited monitoring, and a weak patient safety culture. Stakeholders prioritized improved governance, a national safety reporting system, better health worker training, use of digital tools, and greater patient involvement. These informed a strategy structured around three directions: (1) Leadership and governance, (2) Evidence and innovation, and (3) Literacy and engagement. The final framework comprises 47 prioritized actions, including the creation of regional quality departments, a national patient safety system, updated clinical protocols, and integration of patient-reported outcomes and quality training in medical education.

**Conclusion:**

Greece’s co-created strategy offers a model for other countries aiming to advance whole-system approaches to quality of care through participatory, evidence-informed policymaking. The process not only enhanced relevance and ownership, laying a strong foundation for sustainable implementation, but also assured the use of existing resources to build future directions at national, regional, and local levels. Lessons from Greece’s experience can guide similar efforts to improve quality and safety across diverse health system contexts.

## Introduction

High-quality care and patient safety are global health priorities. World Health Organization (WHO) notes that preventable harm significantly burdens health systems and erodes public trust [1]. One in 20 patients are exposed to preventable harm while 12% of hospitalizations result in serious adverse events or death [2]. Globally, poor-quality care causes over five million deaths annually—more than lack of access—and wastes resources through low-value care and preventable harm, while undermining public trust and worsening inequalities [3, 4]. Poor quality care is often rooted in systemic weaknesses, including inadequate infrastructure, health workforce shortages, insufficient funding, and ineffective governance. Addressing these issues requires strategically planned and coordinated policies to guide reforms and improve care quality and safety across the system [5–7].

Many countries have developed and implemented strategies and action plans to steer improvements in care delivery, safety, and patient experience [8, 9]. Such strategies establish clear priorities and foster accountability, while embedding quality into system-wide norms and practices [10, 11]. In recent years, there has been a growing recognition of the need for comprehensive and inclusive approaches to strategy development, involving diverse stakeholders and drawing upon a range of evidence sources [12]. Inclusivity ensures that strategies are not only technically sound but also reflect values, priorities, and lived experiences of communities they serve [13]. Global initiatives, including WHO’s Global Patient Safety Action Plan 2021–2030, call for all countries to strengthen their quality and safety policies [14].

In recent years, Greece has made progress in specific areas of health system reform, including digitalization, primary care strengthening, and workforce planning, yet the overall quality of care landscape remains fragmented and uneven [15–17]. The absence of an integrated quality policy and governance framework has led to variability in service standards and limited accountability across sectors. Data availability on clinical outcomes, patient experience, and safety events remains scarce, while performance monitoring and feedback mechanisms are underdeveloped [15, 16]. Patient satisfaction surveys, when conducted, show persistent concerns regarding continuity of care, responsiveness, and equity of access [18–20]. National and international analyses highlight several system-level challenges—weak institutional coordination for quality, limited implementation of quality management systems at provider level, insufficient use of data for decision-making, and a culture of safety still in its early stages—all of which underscore the need for a coherent, system-wide strategy to align governance, evidence, and improvement initiatives across the Greek health system. In the context of lacking national quality policy or strategy, the Agency for Quality Assurance in Health S.A. (AQAH S.A.) was established in 2020 and mandated with building a national framework for quality assurance and improvement [21].

In response, the Greek Ministry of Health (MoH)—with support from WHO, AQAH S.A., and the European Commission’s Directorate-General for Structural Reform Support—developed the country’s first National Strategy for Quality of Care and Patient Safety for the 2025–2030 period [22, 23]. This paper documents the development process, key outputs, and lessons learnt—for other countries to use, should they want to apply similar inclusive and evidence-informed approaches. The overall perspective is constructive, acknowledging challenges and limitations while focusing on methodological approaches that contributed to a robust outcome.

## Methods

The strategy was developed through a structured, multi-phase, multi-methods process, prioritizing consultative stakeholder engagement (Table 1) [13, 24]. This approach integrated both quantitative and qualitative data collection, while drawing on core co-creation principles: inclusivity, iterative design, and shared process- and outcome-ownership, to ground the strategy in diverse perspectives and real-world needs [25].

**Table 1.** Overview of the co-creation methodology and timeline for strategy development

Phase	Key activities	Timeframe
Evidence review	Scoping review of international literature and strategies. Situational analysis of the Greek health system.	Mid–late 2023
Needs assessment	Nationwide online survey (*n* = 405). In-depth semi-structured interviews (*n* = 14). Analysis of findings.	May–July 2024 (survey) and June–August 2024 (interviews)
Regional co-creation workshops	Seven in-person workshops across all health regions, with 348 participants engaged across stakeholder groups with prioritization of strategy elements.	March–May 2024
Strategy validation	Drafting of the strategy document. Iterative feedback and stakeholder review. Formal endorsement by the MoH.	Late 2024

Between mid-2023 and late 2024, the research team—comprising staff from WHO Athens Office on Quality of Care and Patient Safety, Greek MoH, AQAH S.A., and academic institutions—designed and coordinated four phases of the strategy development: evidence review, needs assessment, regional co-creation workshops, and strategy development and validation. While broadly sequential, these phases did overlap, such as conducting regional workshops before the completion of the national survey and interviews. The study team included international and national civil servants, policymakers, and researchers with relevant credentials and subject matter expertise. Team members conducting surveys, interviews, and workshop breakout sessions (five women, four men) had previous experience with health strategy development and the Greek healthcare system.

### Evidence review and situational analysis

A scoping review of European and international strategies was carried out, identifying design features, success factors, and implementation considerations relevant to national quality and safety strategies. The review included policy documents, scientific articles, and reports from international organizations and ensured that the strategy development process was informed by the latest evidence and international experience from the onset [26].

Evidence review was complemented by an assessment of Greece’s healthcare system. Policy documents, laws, and existing data on health care performance and outcomes were reviewed, aiming to map current strengths, gaps, and needs—e.g. identifying the absence of a national quality policy, fragmented or siloed safety-improvement initiatives, and areas of suboptimal performance—to tailor the strategy to Greece’s context.

### Needs assessment

To gather system-wide insights, an online survey (*n* = 405) was conducted between May and July 2024, disseminated via health institutions, social media, and WHO/MoH channels. Respondents—clinicians, managers, academics, and patient advocates—from all seven health regions of Greece rated key quality challenges and prioritized improvement areas through structured and open-ended questions. Survey data were analysed descriptively. Frequencies and cross-tabulations were used to identify consensus views and any divergences by stakeholder group.

In parallel, semi-structured interviews (*n* = 14) were conducted with policymakers, regional directors, hospital leads, professional associations, academics, and a national patient association representative, sampled purposefully in consultation with WHO’s stakeholder mapping. Conducted between June and August 2024, interviews followed a common guide and explored systemic challenges, current initiatives, and strategic priorities. Each 60-min interview was recorded with consent. Two researchers thematically coded transcripts using a shared codebook. These qualitative insights added depth to survey findings and informed strategy design [27].

### Regional co-creation workshops

Seven regional workshops were the core of the co-creation process, held from March to May 2024, gathering participants (*n* = 348) from all seven Greek health regions. The objective was to bring stakeholders together to discuss current needs and priorities and to collaboratively shape the elements of the strategy.

Invited by regional authorities, participants included regional and hospital administrators, front-line professionals from hospitals and primary care, representatives of patient associations, and academics from local universities. To ensure inclusiveness, remote access was provided as needed. Each workshop included presentations, breakout sessions, and participatory voting to identify strategic priorities across four thematic areas: governance, safety, clinical guidelines, and engagement. Patient advocates were encouraged to share personal experiences to ground discussions in real-world quality issues. Breakout session inputs were discussed in plenary to identify and vote on key recommendations. Out of 203 eligible participants, 178 participants voted during workshops—excluding lecturers, support staff, and observers—resulting in an 88% response rate. Workshops’ outputs included a set of proposed strategic goals and interventions, and region-specific considerations.

### Strategy validation

Insights from all sources were synthesized, in late 2024, to shape the strategy’s framework. Iterative feedback loops with stakeholders and institutional partners ensured alignment with system needs and implementation feasibility. The project team coordinated an iterative process of feedback and revisions, actively involving AQAH S.A. staff and Greek experts who participated in the regional workshops.

### Stakeholder engagement

Stakeholder engagement and transparency were central to the process, guided by the project’s stakeholder engagement plan. Over 750 individuals contributed through surveys, interviews, workshops, and consultations. Regular meetings were held with MoH leadership to maintain political buy-in, while WHO provided ongoing technical advice to ensure alignment with global quality frameworks. The Greek Patient Association was actively involved throughout, including in all regional workshops and the 1st Athens Patient Forum [28]. The methods deliberately combined quantitative data to broadly gauge perceptions and qualitative insights to explore issues in depth. This triangulation aimed to increase the robustness of the findings and the credibility of the strategy’s content.

### Ethical considerations

The strategy development project was carried out as a policy and health system strengthening initiative. All participants in surveys, interviews, and workshops provided informed consent for use of their inputs in formulating the strategy. Ethical approval for the needs-assessment component was granted by the Bioethics Committee of the School of Medicine, Faculty of Health Sciences, Aristotle University of Thessaloniki, Greece (Decision No. 202/2024).

## Results

### Evidence review and situational analysis

The scoping review identified key success factors from international quality strategies, including stakeholder engagement, clear governance, patient-centredness, and integrated monitoring [26]. Table 2 outlines how these were reflected in Greece’s strategy. The policy and data situational analysis identified a number of longstanding systemic challenges, including fragmented service delivery, limited patient-centredness, variations in quality standards across settings, and the lack of a unified national policy framework.

**Table 2.** Scoping review-identified international best practices and ‘best buys’ informing evidence-based policy development and implementation

Theme	Summary of the evidence	Greek strategy application
**Design and development**	A comprehensive stakeholder consultation process involving government bodies, health-care providers, regional/national authorities, and patient partnerships.	Extensive co-creation process, including nationwide needs assessment (405 survey responses and 14 interviews), and seven health region workshops involving over 750 participants. Design driven by inclusivity and ownership at all system levels.
**Target populations**	Health workers and patients are the top target populations. Administrators and policymakers are also addressed.	Strategy focuses on health workers (training, leadership development, well-being) and patients (engagement, rights, health literacy). Administrators and system-level actors are also a key audience through governance reforms and monitoring structures.
**Priority areas**	Patient safety; quality measurement and continuous improvement; people-centred care; transparency; workforce safety; and learning systems.	Strategic framework structured around three directions and encompassing 11 objectives and 47 actions, including national patient safety reporting system, use of clinical guidelines, and digital tools to drive quality.
**Enablers of implementation**	Leadership commitment and support; stakeholder collaboration and coordination; workforce well-being.	Institutionalized governance and multisectoral coordination mechanisms developed. National training to build capacity, promote collaboration, and support workforce retention and motivation.
**Barriers to implementation**	Common obstacles include resource constraints and resistance to change.	Needs assessment confirmed similar issues. Strategy includes phased roll-out with quick wins and long-term reforms; efforts to secure financial sustainability through alignment with broader national health investments.
**Evaluation and monitoring**	Data collection and transparency are essential, but many strategies lack clear national indicators or aligned monitoring tools.	Strategy defines a national monitoring framework with core indicators linked to the strategic objectives. Plans for digital platform integration, use of patient-reported measures, and public dissemination of results to promote accountability and trust.

### Needs assessment

The national survey and stakeholder interviews revealed a strong convergence on system weaknesses and priority needs. Respondents flagged workforce shortages, lack of protocols, and communication issues as major barriers. Approximately 60% rated national quality governance as ineffective, and over 70% described patient safety systems as underdeveloped [27]. Digital tools and structured reporting were seen as underutilized [27].

In-depth interviews reinforced these findings and provided a more nuanced perspective on systemic challenges, emphasizing fragmented governance, lack of formal accountability, and a prevailing culture of blame that discouraged incident reporting. Table 3 presents a synthesis of the quantitative and qualitative findings across four domains: system barriers, clinical effectiveness, safety culture, and patient engagement.

**Table 3.** Key findings from the national needs assessment^a^

Domain	Key findings
**Quality of care and patient safety: perceptions, enablers, and barriers**	Participants rated the current quality of care and patient safety in Greece as moderate (mean scores: 5.7 and 5.6 out of 10, respectively). Fewer than half believed national policies align with international standards. Top barriers identified were inadequate patient-to-staff ratios (48%), insufficient funding (33%), and limited training opportunities (13%). Digital health technologies were widely viewed as promising enablers.
**Clinical effectiveness and reporting culture**	A significant majority indicated the absence of a functional national adverse event reporting system. The main deterrents to reporting were fear of blame (49%) and scepticism about the impact for change of follow-up actions (27%). Safety audits were reported to occur infrequently or not at all (48%). Key improvement priorities for clinical guidelines included antibiotic prescription, infection control, referral criteria, and noncommunicable diseases.
**Learning culture and workforce development**	Training on quality and safety was perceived as insufficient (mean score: 5.4/10 for both areas). Practical, hands-on training and interprofessional education were considered highly effective (mean scores: 8.6–8.8/10). Reported barriers included lack of leadership support (30%), time constraints (21%), and limited training resources (21%).
**People-centredness and patient engagement**	Patient involvement in care planning was rated low (4.7/10), and perceived health literacy levels were similarly modest (4.4–4.5/10). Participants stressed the need to strengthen patient education and empower citizens. Digital tools (e.g. apps, portals) were seen as effective channels for improving health literacy (7.1/10). Key barriers included the absence of structured health education, a dominant doctor-centred culture, and difficulties accessing reliable information.

a Quantitative data in the table represent average scores on a 10-point Likert scale (1 = very poor, 10 = excellent), calculated as the mean across all responses for each item. Where applicable, percentage values refer to the proportion of survey participants who selected a given option in multiple-choice questions. Qualitative findings, particularly in the learning culture and people-centredness domains, were derived from thematic content analysis of open-ended survey responses and interviews.

### Regional co-creation workshops

Seven workshops with 348 participants generated actionable insights and helped validate the assessment results. In terms of leadership and governance strengthening, establishing regional quality committees to oversee implementation and accountability was seen as a priority. So was empowering AQAH S.A. with expanded mandates to monitor and enforce national standards. To improve incident reporting and safety systems, developing a national patient safety reporting system, including patient-reported incidents, to standardize learning from adverse events, was proposed. Stakeholders stressed the need for a non-punitive feedback culture to encourage reporting. When discussing health workforce training, the integration of quality and safety training into medical and nursing curricula, as well as mandatory annual safety training for all healthcare professionals, including frontline staff, was suggested [29]. To enhance patient and public engagement, development of hospital-based patient advisory councils to amplify patient voices in service design was proposed. So was launching health literacy campaigns to educate the public on quality care standards. Finally, to leverage on digital health solutions and tools, it was recommended to expand the use of electronic health records, also by integrating quality indicators into routine reporting. Finally, the development of a ‘National Quality Dashboard’ to track real-time performance data on both the system- and facility-level was proposed.

Despite consistent priorities across different stakeholder groups and regions, workshops also uncovered some stakeholder- and region-specific barriers and needs. For instance, some clinicians initially placed less emphasis on patient engagement, whereas patient groups obviously highlighted it. Hospital managers emphasized financing and staffing issues as preconditions for quality. Workshops in urban areas (e.g. Athens, Thessaloniki) prioritized data-driven governance and hospital-based safety systems. On the other hand, workshops in rural and island regions (e.g. Aegean, Crete) prioritized telemedicine expansion, workforce retention, and community-based care models.

Some participants, especially front-line clinicians, were initially sceptical about another strategy or plan that might not be fully implemented. However, the co-creation approach helped prevent consultation fatigue and cynicism, with many participants expressing appreciation for being heard for the first time on these issues. In the final validation stage, stakeholders could witness their influence on the strategy content. This level of engagement helped turn some sceptics into advocates. Additionally, EU and WHO involvement increased confidence in implementation support.

### Strategy validation and final framework

The final strategy, shaped by these findings, includes a vision, three strategic directions, 11 objectives, and 47 prioritized actions. A vision of ‘a health care system where quality is a daily commitment, ensuring that all individuals trust that health care will be safe, respectful, equitable and efficient’ was informed by both technical inputs and patient voices, with terms such as ‘safety’, ‘respect’, ‘dignity’, and ‘efficiency’ emerging prominently. Strategy’s three strategic directions—(1) Leadership and governance, (2) Evidence and innovation, and (3) Literacy and engagement, with objectives under each—are accompanied by a series of actions and monitoring indicators. Table 4 presents on overview of the strategy, from its vision through the three strategic directions to 11 objectives and accompanying tracer indicators.

**Table 4.** Greek National Strategy for Quality of Care and Patient Safety (2025–2030) overview^a^

Vision	A health care system where quality is a daily commitment, ensuring that all individuals trust health care to be safe, respectful, equitable, and efficient.	
Strategic directions	Objectives	Tracer indicators
**1. Leadership and governance**	1.1. Strengthen the legal provisions for quality of care and patient safety	Quality of care structures established at the regional and local level Data are digitally collected to inform national quality of care indicators
	1.2. Implement a three-level governance model for quality of care and patient safety	
	1.3. Enhance efficiency, quality of care, and workforce well-being	
	1.4. Orient health system towards data-driven decision making processes	
**2. Evidence and innovation**	2.1. Adopt standards for health care providers	Increase compliance with national protocols Reduce avoidable harm at all care levels Train health workers in quality of care
	2.2. Consolidate evidence-based and safe clinical practices	
	2.3. Foster a continuous improvement, safety and learning culture	
	2.4. Enable health workforce skills in quality of care and patient safety	
**3. Literacy and engagement**	3.1. Enhance public participation in the health system	Results from patient experience surveys are published Patient representatives included in governance structures Massive online open courses used by the public
	3.2. Empower patients, caregivers, and families	
	3.3. Learn from people’s perspectives	

a Source: Adapted from Agency for Quality Assurance in Health S.A. [30].

During the process, questions were raised about how the strategy would be implemented given resource constraints. Some participants feared that overly ambitious goals, like nationwide accreditation or extensive training programs, might be unrealistic in the short term. Final version of the strategy tackled this through priority and phased actions, supported by existing resources and those requiring new investments or long-term efforts.

To support sustainability and track progress, the strategy also includes a dedicated implementation and monitoring framework. Alongside priority actions with defined timelines and responsibilities, the strategy additionally incorporates national-level monitoring mechanisms, such as tracer indicators linked to strategic directions, national quality-of-care dashboard, and periodic reporting tools. Institutional capacity-building, notably through AQAH S.A. and regional quality departments, is expected to further strengthen system-wide commitment to continuous quality improvement and long-term impact.

The Greek MoH approved the strategy in late 2024 and it launched publicly in early 2025.

The complete National Strategy for Quality of Care and Patient Safety (2025–2030) is publicly available on the official website of AQAH S.A. [30]. Supplementary File S1 presents the accompanying strategy brochure, summarizing its main objectives and actions for a general audience.

## Discussion

### Statement of principal findings

The development of the National Strategy for Quality of Care and Patient Safety for Greece 2025–2030 represents a significant step toward strengthening healthcare quality and safety. Co-created through a structured, participatory approach involving over 750 stakeholders nationwide, the strategy explicitly addresses longstanding challenges such as fragmented governance, weak monitoring, limited training, and poor patient engagement. The resulting framework, comprising vision, three strategic directions, 11 objectives, and 47 actions, offers a roadmap aligned with system needs and stakeholder expectations. Its key strength is the co-creation methodology used, fostering broad ownership and consensus among patients, providers, and policymakers.

### Strengths and limitations

The strategy was developed using a robust multi-method approach, incorporating surveys, interviews, workshops, and international evidence. This allowed for triangulation and a holistic understanding of quality gaps. The scoping review helped anchor the strategy in international best practices, while the national needs assessment and regional workshops provided insights into local gaps and priorities. The combination of structured survey data and thematic qualitative insights contributed to the strategy’s credibility and relevance. A key strength of the process was its participatory and multi-level nature. Stakeholder engagement extended from central policy actors to front-line workers and patients and from urban centres to remote regions. This generated shared understanding, ensuring the strategy reflects real system needs, not just aspirations. Patient voices helped shape the language and values of the vision statement, while clinicians and administrators contributed to identifying realistic, implementable actions.

Likewise, limitations must be acknowledged. The needs assessment survey used non-probability sampling, distributed through networks and platforms, and might not ensure representativeness across all health regions or professional categories. Equally, self-reported data may be affected by social desirability bias. Interviews, though purposive and diverse, captured a limited number of perspectives. Workshop participants were known to and nominated by regional health authorities, which might have introduced selection bias. These risks were mitigated by using multiple data sources, anonymous survey administration, and thematic validation across workshops. Overall, the process strengthened the credibility and relevance of the strategy’s content.

### Interpretation within the context of the wider literature

Globally, the development of national quality and safety strategies has become a cornerstone of health-system strengthening. The WHO Handbook for National Quality Policy and Strategy (2018) and the Global Patient Safety Action Plan 2021–2030 both stress inclusive, system-wide approaches linking evidence generation, stakeholder participation, and implementation accountability [10, 14]. Recent literature confirms that strategies for high-quality health systems align with these principles, by combining multisectoral participation, iterative validation, and explicit monitoring frameworks [4, 8, 26, 31].

The Greek experience contributes new empirical evidence on how such principles can be operationalized through structured co-creation at national scale and stands out for its geographical inclusiveness, process transparency, and alignment with WHO and OECD guidance. The co-creation approach also addressed implementation scepticism—common in systems with past strategies that failed to materialize. The Greek strategy development process resonates with international lessons that emphasize how early and continuous stakeholder engagement strengthens legitimacy and feasibility, while structured validation improves implementability [26, 32, 33].

Strategy’s structure—three strategic directions focused on governance, evidence, and engagement—mirrors global quality frameworks but also responds directly to Greece’s national context. Governance gaps were addressed by proposing national and regional quality committees and strengthening AQAH S.A.’s mandate. Evidence-based practice was advanced through actions such as standardizing guidelines and safety protocols, improving data infrastructure, and expanding use of outcome indicators. Patient engagement and literacy were prioritized through initiatives such as advisory councils, public campaigns, and inclusion of PROMs and PREMs. These findings support the broader proposition that co-creation is not only normatively desirable but pragmatically associated with greater ownership and route-to-implementation [34–36].

Compared to strategies in other European countries, the Greek approach placed greater emphasis on early national–regional alignment. In Italy, regional autonomy has made strategy implementation uneven, while Portugal has focused heavily on national-level observatories [37, 38]. Greece’s extensive regional workshops aimed to reduce such disconnects by involving health professionals, managers, and patients in co-designing interventions appropriate for their specific settings.

The added value of the co-creation approach appears 2-fold. On one hand, it improves the robustness of the strategy document—through richer, context-sensitive content. Additionally, co-creation enhances the likelihood of implementation by fostering legitimacy and shared process and product ownership. Evidence from other systems suggests that strategies developed through participatory design are more sustainably embedded in national governance frameworks and accountability processes [26, 34, 35]. Future research may be needed to bring insights through longitudinal evaluation of strategy uptake, institutionalization of quality of care whole-system governance, and measurable improvements in outcomes that matter to individuals and populations. Key challenges in the strategy development process included balancing inclusiveness with efficiency and managing expectations amid resource constraints. Early phases underscored the need to sustain participation and confidence among stakeholders. Maintaining participation required clear communication of how feedback shaped successive drafts—an aspect consistent with literature on sustaining engagement in co-creation [35, 36]. In retrospect, the process might have benefited from even earlier involvement of patients and healthcare professionals in agenda-setting and from a more structured impact-assessment plan defined at the outset.

### Implications for policy, practice, and research

Experience from Greece provides transferable insights for countries developing or updating national quality of care and patient safety policies and strategies. The process highlights several interdependent factors that contributed to the credibility and planned sustainability of the resulting strategic framework.

Sustained engagement and involvement were foundational. High-level political commitment, in combination with ongoing technical support from WHO, created legitimacy and policy momentum, while inclusive participation through open calls, regional workshops, and iterative feedback loops fostered professional trust and ownership. Transparent communication on how feedback was used helped sustain motivation and constructive participation over time, a common challenge in large-scale policy development and reform implementation processes [9, 35]. This also ensured that stakeholders perceived the strategy as a shared product rather than an externally driven development.

Equally critical was the integration of evidence synthesis with participatory deliberation, which strengthened the technical and contextual coherence of the strategy. The Greek process combined scoping reviews, a nationwide needs assessment, and regionally focused workshops, enabling simultaneous top-down and bottom-up alignment. This multi-methods approach helped bridge scientific rigour with implementation feasibility, a consideration highlighted in the WHO Handbook for National Quality Policy and Strategy (2018) and reflected in comparative experiences from other countries [9, 10, 31]. Such triangulation of data and stakeholder insights enhanced confidence that the proposed actions were both evidence-based and operationally viable within existing resources.

Another important lesson concerns inclusiveness and perspective diversity. Early engagement of patient associations, professional societies, and subnational authorities cultivated a shared sense of accountability and supported cross-regional consistency in priorities. Similar findings from evaluations of co-creation approaches indicate that diverse stakeholder representation enhances legitimacy and long-term adoption of quality-improvement initiatives [34, 35]. In Greece, these mechanisms transformed engagement from tokenistic consultation into a practical partnership, setting the foundation for continued collaboration during implementation.

Together, these factors suggest that co-creation functions not only as an inclusive practice ideal but also as a pragmatic approach for ensuring sustainable quality-assurance and improvement efforts. High-level commitment, contextualized evidence-informed content development, and inclusive engagement improve the substantive quality of strategic documents while simultaneously strengthening the institutional and social foundations required for their successful implementation.

While international guidelines help, successful strategies must be tailored to local realities—health system structure, culture, and resources. The process did not stop at writing the document. By defining responsibilities, timelines, and resource needs, the strategy aims to avoid the pitfalls of previous reform efforts that lacked execution follow-through. Detailed roadmap adapts the national strategy to varied regional and local capacities. Aligning strategic actions with existing EU-funded programmes and national investments further strengthens feasibility, while planned phased rollout and ‘quick wins’ boosted stakeholder confidence.

Future research should examine whether co-creation processes are associated with stronger uptake of strategic actions, sustained governance arrangements, and measurable improvements in quality of care and patient safety outcomes. Longitudinal evaluation of strategy implementation across different contexts could also generate valuable insights on the mechanisms through which co-creation enhances ownership and long-term system change. Comparative studies between countries or regions using similar co-creation methods could also help refine and expand this approach.

The transferability of the Greek model to other contexts should be considered in light of potential influencing factors, such as the cultural context, health system structure, and resources. The specific cultural values and norms within a healthcare system can influence attitudes towards quality and safety and the implementation of improvement initiatives. Differences in healthcare system organization, financing, and delivery can affect the applicability of certain strategies and interventions.

## Conclusions

The development of the National Strategy for Quality of Care and Patient Safety for Greece 2025–2030 demonstrates the value of a multi-methods approach and stakeholder engagement in creating effective and sustainable national strategies. The Greek experience underscores the value of context awareness, inclusive engagement, and comprehensive methods to address quality and safety challenges. Beyond the technical output, the process itself strengthened legitimacy, ownership, and feasibility, key determinants of sustainable implementation. The strategy’s focus on leadership, evidence-based practice, and patient engagement provides a strong foundation for achieving its ambitious goals. Phased implementation and continued monitoring and evaluation will be crucial to assess the strategy’s impact and ensure its long-term success.

## Supplementary Material

mzaf135_Supplementary_Data

## Data Availability

The data generated or analysed during this study are available from the authors on reasonable request and with permission from WHO and the Greek MoH.
