# Supplementary material for: Development of the National Strategy for Quality of Care and Patient Safety for Greece: co-creation process and lessons learnt
Source: Int J Qual Health Care. 2025 Dec 30;38(1):mzaf135. doi: 10.1093/intqhc/mzaf135 (PMC12854083; doi:10.1093/intqhc/mzaf135)
Supplement: mzaf135_Supplementary_Data [file mzaf135_supplementary_data.zip › Supplement - COREQ Checklist.docx]

**Appendix:** Consolidated Criteria for Reporting Qualitative Research (COREQ) Checklist^a^

| **#** | **Item** | **Question/description** | **Answer** | **Section** |
| --- | --- | --- | --- | --- |
| **Domain 1: Research team and reflexivity** | | | | |
| **Personal Characteristics** | | | | |
| 1 | Interviewer/ facilitator | Which author/s conducted the interview or focus group? | VRF, CV, MS, CT, AM, VP, ZT and VK. | Methods |
| 2 | Credentials | What were the researcher’s credentials? | VRF: MD, PhD; CV: MSc; MS: MSc; CT: RN, MSc; AM: MSc; VP: MSc; ZT: MD, MSc, PhD; VK: MSc, PhD; DK: RN PhD | Methods and authors’ list |
| 3 | Occupation | What was their occupation at the time of the study? | Researchers were international and national civil servants, policymakers and researchers with relevant credentials and subject matter expertise. | Methods |
| 4 | Gender | Was the researcher male or female? | Five women and four men. | Methods |
| 5 | Experience and training | What experience or training did the researcher have? | The research team included researchers and practitioners with previous experience of working with health data and policy, designing health system-related strategies and the experience of working in the Greek health system, as well as with health systems internationally. | Methods |
| **Relationship with participants** | | |  |  |
| 6 | Relationship established | Was a relationship established prior to study commencement? | Participants were informed in advance about the study purpose; some had prior collaboration with the WHO or Ministry. | Methods |
| 7 | Participant knowledge of the interviewer | What did the participants know about the researcher? e.g. personal goals, reasons for doing the research. | Participants knew the study aimed to inform national strategy; introductions included researcher affiliation and purpose. | Methods |
| 8 | Interviewer characteristics | What characteristics were reported about the interviewer/facilitator? e.g. Bias, assumptions, reasons and interests in the research topic | Researchers were introduced by role and affiliation; biases not explicitly discussed. | Methods |
| **Domain 2: study design** | | |  |  |
| **Theoretical framework** | | |  |  |
| 9 | Methodological orientation and Theory | What methodological orientation was stated to underpin the study? e.g. grounded theory, discourse analysis, ethnography, phenomenology, content analysis | Descriptive qualitative and thematic analysis; content analysis for open-ended survey data. | Methods |
| **Participant selection** | | |  |  |
| 10 | Sampling | How were participants selected? e.g. purposive, convenience, consecutive, snowball | Purposive sampling based on stakeholder mapping for interviews; regional representation for workshops; broad outreach for survey. | Methods |
| 11 | Method of approach | How were participants approached? e.g. face-to-face, telephone, mail, email | Survey via institutional and social media channels; interviews arranged via email/phone; workshop invites through regional authorities. | Methods |
| 12 | Sample size | How many participants were in the study? | Survey: 405; Interviews: 14; Workshops: 348 participants across 7 regions. | Methods |
| 13 | Non-participation | How many people refused to participate or dropped out? Reasons? | Not relevant for the current manuscript; except for the stated 88% response rate during the regional stakeholder workshop group work voting. | Methods |
| **Setting** | | | | |
| 14 | Setting of data collection | Where was the data collected? e.g. home, clinic, workplace | Online (survey); virtual or in-person (interviews/workshops). | Methods |
| 15 | Presence of non-participants | Was anyone else present besides the participants and researchers | Workshops included observers and support staff, not in breakout groups. | Methods |
| 16 | Description of sample | What are the important characteristics of the sample? e.g. demographic data, date | Stakeholders from 7 regions; policymakers, providers, academics, and patient representatives. | Supported in Methods and Results |
| **Data collection** | | | | |
| 17 | Interview guide | Were questions, prompts, and guides provided by the authors? Was it pilot tested? | Yes; semi-structured guide developed; not piloted. | Methods |
| 18 | Repeat interviews | Were repeat interviews carried out? If yes, how many | No repeat interviews. | NA |
| 19 | Audio/visual recording | Did the research use audio or visual recording to collect the data? | Yes; interviews recorded with consent. | Methods and Ethics |
| 20 | Field notes | Were field notes made during and/or after the interview or focus group? | Yes—transcripts were coded by two researchers. | Methods |
| 21 | Duration | What was the duration of the interviews or focus groups? | ~60 minutes for interviews; workshops half or full day. | Methods |
| 22 | Data saturation | Was data saturation discussed? | Not explicitly discussed. | NA |
| 23 | Transcripts returned | Were transcripts returned to participants for comment and/or correction? | No transcript review; feedback was obtained via validation steps. | Methods |
| **Domain 3: analysis and findings** | | | | |
| **Data analysis** | | | | |
| 24 | Number of data coders | How many data coders coded the data? | Two researchers coded transcripts using a shared codebook. | Methods |
| 25 | Description of the coding tree | Did authors provide a description of the coding tree? | No included in the manuscript. | NA |
| 26 | Derivation of themes | Were themes identified in advance or derived from the data? | Themes derived from the data. | Methods |
| 27 | Software | What software, if applicable, was used to manage the data? | Excel. | Not specified in text |
| 28 | Participant checking | Did participants provide feedback on the findings | Stakeholder feedback during validation phase; no formal member-checking. | Methods |
| **Reporting** | | | | |
| 29 | Quotations pr­esented | Were participant quotations presented to illustrate the themes/findings? Was each quotation identified? e.g. participant number | No. | NA |
| 30 | Data and findings consistent | Was there consistency between the data presented and the findings? | Yes; findings aligned with data sources. | Results |
| 31 | Clarity of major themes | Were major themes clearly presented in the findings? | Yes; governance, safety, training, and engagement are clear. | Results and Discussion |
| 32 | Clarity of minor themes | Is there a description of diverse cases or discussion of minor themes? | Yes; regional differences, scepticism, and patient engagement nuances discussed. | Results and Discussion |

^a^ Tong A, Sainsbury P, Craig J. Consolidated criteria for reporting qualitative research (COREQ): a 32-item checklist for interviews and focus groups. Int J Qual Health Care 2007;19:349–57.
