# Supplementary material for: Development of the National Strategy for Quality of Care and Patient Safety for Greece: co-creation process and lessons learnt
Source: Int J Qual Health Care. 2025 Dec 30;38(1):mzaf135. doi: 10.1093/intqhc/mzaf135 (PMC12854083; doi:10.1093/intqhc/mzaf135)
Supplement: mzaf135_Supplementary_Data [file mzaf135_supplementary_data.zip › Supplementary File S1.pdf]

# National Strategy for Quality of Care and Patient Safety for Greece
# 2025 – 2030

## Why do we need a Strategy for Quality of Care and Patient Safety in Greece?

Health care worldwide often faces significant challenges related to quality, resulting in unnecessary suffering, loss of trust, and substantial economic costs. This is the first-ever National Strategy for Quality of Care and Patient Safety in Greece to address these gaps and enhance governance, accountability, and the quality of care through a unified, strategic approach.

## How did we design this Strategy?

Through collaboration between the WHO Office on Quality of Care and Patient Safety in Athens, the Ministry of Health of Greece, ODIPY, and the European Commission's Directorate-General for Structural Reform Support (DG REFORM). Using a mixed-method approach that includes literature reviews, situation analyses, surveys, interviews, and a co-creation process.

### Input

- Literature review of best internation practices
- Situation analysis oncurrent practices in Greece
- Semi-structured interviews of opinion leaders
- Open online survey to capture needs and priorities

### Co-creation proccess

- Greek Health Region Workshops
- Iterative design, aligment and draft

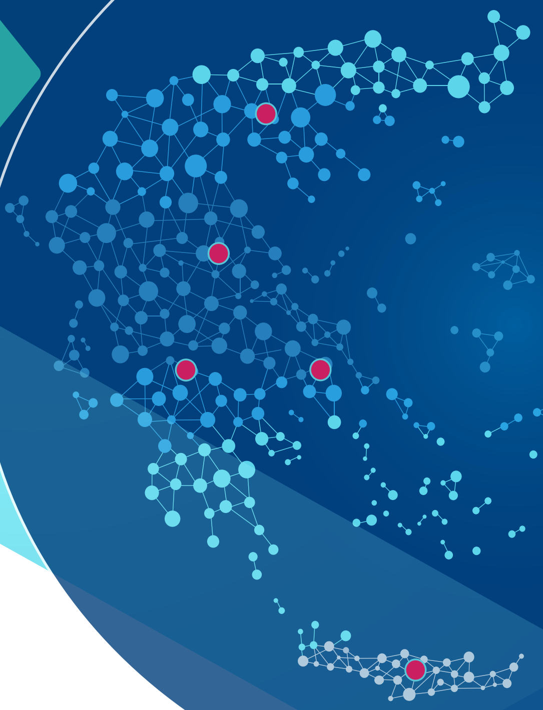

**1st Health Region**
Athens

**2nd Health Region**
Athens (Pireaus)

**3rd Health Region**
Thessaloniki

**4th Health Region**
Thessaloniki

**5th Health Region**
Larissa

**6th Health Region**
Patra

**7th Health Region**
Heraklion

Seven workshops across Greece, engaging 348 participants. Discussions facilitated by both Greek and international experts, along with the expresineces shared by 14 different countries. Insights from 405 participants in an online survey and 7 workshops across all 7 health regions in Greece with 348 stakeholders, including patients and patients' organizations, shaped actionable recommendations towards: quality of care, patient safety, and patient engagement in healthcare provision in Greece.

World Health Organization
European Region

Funded by the European Union

HELLENIC REPUBLIC
Ministry of Health

odipy
AGENCY FOR QUALITY ASSURANCE IN HEALTH

# VISION

A healthcare system where quality is a daily commitment, ensuring that all individuals trust healthcare to be safe, respectful, equitable, and efficient.

## Our approach

The Strategy aims to achieve three main goals through three strategic directions, including 11 objectives and 47 prioritized actions, which are designed for phased, stepwise implementation. These actions focus on improving patient safety, governance, clinical guidelines, and health literacy across all levels of the healthcare system.

### Leadership & governance

To nurture an efficient, accountable, transparent and data-driven health system

- Strengthen the legal provisions for quality of care and patient safety
- Implement a three-level governance model for quality of care and patient safety
- Enhance efficiency, quality of care, and workforce well-being
- Orient health system towards data driven decision making processes

### Evidence & innovation

To foster trust to an effective, safe and equitable health system

- Adopt standards for healthcare providers
- Consolidate evidence-based and safe clinical practices
- Foster a continuous improvement, safety and learning culture, also by Develop and deploy a national voluntary patient safety incident electronic reporting system
- Enable health workforce skills for quality of care and patient safety

### Literacy & engagement

To create patient partnershipsin health care provision

- Enhance people participation in the health system
- Empower patients, caregivers, and families
- Learn from peoples' perspectives
